# Supplementary material for: WordCommentsAnalyzer: A windows software tool for qualitative research
Source: F1000Res. 2018 Sep 4;7:536. Originally published 2018 May 3. [Version 2] doi: 10.12688/f1000research.14819.2 (PMC6137411; doi:10.12688/f1000research.14819.2)
Supplement: Supplementary file 3 [file f1000research-7-17614-s0002.tgz › b2a79496-4ada-47c4-9682-a99ce867836d.zip!/Eight Iranian Health Journals/Yafteh _57.docx]

© Yafteh  . This work is licensed under CC BY 4.0 (https://creativecommons.org/licenses/by/4.0/).

Volume & Issue: دوره 6 - شماره 4

Title: تأثیر مصرف نان کامل حاصل از گندم غنی شده با کودهای میکرو بر روی سرم

http://yafte.lums.ac.ir/article-1-942-fa.html

مقدمه: نظر به اهمیت کمبود روی و تلاش برای یافتن راه حل های مناسب جهت مبارزه با آن و به منظور تعیین اثر مصرف نان کامل حاصل از گندم غنی شده با کودهای میکرو بر روی سرم این مطالعه در سال 1381 انجام گرفت. مواد و روشها: این تحقیق به صورت کارآزمایی صحرایی دو سو کور در دو روستای تجرق و خانقاه(تجربی و شاهد) از توابع شهرستان میانه در استان آذربایجان شرقی انجام گرفت.تعداد 350 نفر از گروههای مختلف سنی و جنسی از هریک از روستاهای مورد بررسی بر اساس نمونه گیری تصادفی انتخاب شدند. 257 نفر از روستای تجرق و 268 نفر از روستای خانقاه در این مرحله شرکت نمودند. بررسی مصرف غذایی با روش 24 ساعت یاد آمد (یک روز) و دو روز ثبت مواد غذایی برای هر نمونه انجام شد. 5 سی سی خون از هر فرد اخذ شد. اندازه گیری روی سرم با روش جذب اتمی برای هر یک ازنمونه ها به عمل آمد. 50 تن گندم حاصل از خاک غنی شده با کودهای میکرو به آرد کامل تبدیل گردید و به مدت 4 ماه به اهالی روستای تجرق داده شد. اهالی روستای خانقاه نیز آرد کامل حاصل از گندم معمولی را در همین مدت دریافت نمودند. سپس روی سرم مجددا اندازه گیری شد. تعداد افراد شرکت کننده در این مرحله 160 نفر در روستای تجرق و 150نفر در روستای خانقاه بود. یافته ها: میزان آهن و روی موجود در نانهای غنی شده و معمولی (شاهد) تفاوت معنی دار داشت (01/0p<). نسبت مولی اسید فیتیک به روی در نان غنی شده کمتر از نان شاهد بود ولی نسبت مولی آهن به روی و برعکس در دو نوع نان تفاوتی نداشت. روی دریافتی در گروههای شاهد و مورد باهم تفاوت معنی داری داشت؛اما آهن دریافتی تنها در گروه های سنی مردان و زنان60-13سال باهم تفاوت معنی دار داشت و در سایر گروهها تفاوتی مشاهده نشد.روی سرم در همه گروههای سنی روستای تجربی و شاهد(بجز مردان) افزایش یافت(01/0 p<سنی). نتیجه گیری: به نظر می رسد برای تولید گندم مناسب باید ترکیب عناصر در کود به صورتی که تداخلی بین آنها ایجاد نشود اصلاح گردد ونسبت مولی اسیدفیتیک به روی نیز به زیر 15 وحتی پایین ترکاهش یابد.

Title: نقش فاکتور های مؤثر در پیدایش عوارض اندام تحتانی بعد از برداشت ورید صافن در عمل جراحی گرافت شریان کرونری

http://yafte.lums.ac.ir/article-1-944-fa.html

مقدمه: عوارض زخم پا بدنبال اعمال جراحی گرافت شریان کرونری در نوشته هاکمتر وبه ندرت گزارش شده است.در این مقاله تجربه خود را دردرمان30بیمار مبتلا به عارضه زخم پابعد از عمل گرافت شریان کرونری ارائه میدهیم. مواد و روشها: در این مطالعه گذشته نگر2100پرونده بیماران تحت عمل جراحی گرافت شریان کرونری با استفاده از ورید صافن در مدت بیش از 5 سال ودر دو مرکزمورد بررسی قرار گرفتند. ریسک فاکتورهای مهم برای آنهایی که دچار زخم پا شده بودندمورد آنالیز قرار گرفتند و با تمام بیمارانی که درهمان زمان تحت عمل جراحی گرافت شریان کرونری قرار گرفته بودندمقایسه شدند. یافته ها: یافته ها نشان داد30بیمار بعد از عمل گرافت شریان کرونری؛ دچارعارضه زخم اندام تحتانی بعد از عمل شدند(4/1درصد) که 19نفر از آنهانیاز به عمل جراحی داشتندو11نفراز بیماران تحت درمان دارویی قرار گرفتند.اقدامات درمانی شامل؛6نفر دبریدمان زخم؛ 2نفر گرافت پوست؛1نفر عمل جراحی آنژیو پلاستی؛7مورد فاشیوتومی؛ 1موردآمبولکتومی وفاشیوتومی و 2نفر انتقال بافت آزادبود.از14متغیر ارزیابی شده با آنالیزچندگانه؛زمان بای پس قلبی؛ مدت عمل جراحی وزدن بالون داخل شریان بعداز عمل به عنوان متغیر های مستقل معنی دار مشخص شدندکه درایجادعارضه زخم پا دخالت داشتند(p<0.05). نتیجه گیری: عوامل ایجاد کننده زخم پا بعداز برداشت وریدصافن در اعمال جراحی گرافت شریان کرونری چند گانه هستند. برای کاهش این عارضه توصیه میشودقبل از برداشت ورید صافن در اعمال جراحی گرافت شریان کرونرارزیابی عروقی اندام انجام گردد؛ به مناسب بودن تکنیک عمل توجه شود ومحل برداشت وریدبطور دقیق انتخاب گردد.

Title: مقایسه استانداردهای بیمارستانی با اصول ایزو و ارائه الگوی مناسب برای تدوین استاندارد بیمارستانی

http://yafte.lums.ac.ir/article-1-945-fa.html

مقدمه: استانداردهای بیمارستانی یکی از با ارزش ترین عناصر مفهومی در سازمان و مدیریت بیمارستان به شمار می رود. هدف از انجام پژوهش این بود که از طریق مقایسه، کاستی های نحوه فعلی ارزشیابی بیمارستان ها را نسبت به آنچه که استانداردهای کیفیت مدیریت ایزو مطرح می کند، شناسایی کرده و بر اساس یافته های حاصل الگویی را برای تدوین استانداردهای بیمارستانی پیشنهاد نماید. مواد و روشها: پژوهش حاضر مطالعه ای توصیفی- تطبیقی است که برای مقایسه استانداردهای بیمارستانی،کتابچه «ب» ارزشیابی بیمارستان ها، با اصول ایزو 9000 نسخه 1994 انجام شده است. در مرحله اول، تحلیل نظری و مقایسه گزاره های کتابچه با الزامات ایزو انجام شده؛ سپس با استفاده از روش بارش افکار برای تعیین فهرست ها، روش رای گیری متعدد و تکنیک گروه اسمی برای گزینش و روش های فرآیند تحلیل سلسله مراتب(AHP) و ماتریس انتخاب برای اولویت بندی و وزن دهی به گزینه ها، الگوی 8 مرحله ای برای تدوین استانداردهای بیمارستانی ارائه گردید. با استفاده از روش شبه دلفی الگوی ارائه شده مورد تایید قرار گرفت. یافته ها: در کتابچه «ب» مجموعا 853 سوال از نظر تطابق با اصول ایزو مورد بررسی قرار گرفتند و در مقابل 853 گزاره کتابچه، 1026 بار عناصر ایزو آورده شد. در این تطبیق، بندهای " ممیزی های داخلی کیفیت"، " کنترل فرآیند" و " بازرسی و آزمایش" از استانداردهای ایزو 9000 نسخه 1994 به ترتیب 339 و 302 و 158 بار تکرار شدند که در مجموع 8/77% از عناصر نوشته شده را شامل می شود. و 17 بند دیگر فقط 2/22% از کل دفعات تکرار عناصر ایزو را تشکیل می دادند که از این تعداد 7 بند اصلا مصداقی در « کتابچه ب » نداشتند و 5 بند دیگر نیز کمتر از 7 مصداق (از 1010 سوال) را داشتند. نتیجه گیری: طرز نگارش گزاره های ارزشیابی در « کتابچه ب » با آنچه که در ممیزی ایزو انجام می شود، تفاوت فاحش دارد. یافته های تحقیق نشان می دهد که علیرغم جهش مثبتی که تدوین « کتابچه ب » در ارزشیابی بیمارستان های عمومی کشور داشت، به دلیل فقدان تجربه کافی در استاندارد نویسی مبتنی بر اصول ایزو، نقصان های جدی در ارزشیابی کامل از بیمارستان، به نحوی که جامع و یکپارچه سیستم و مدیریت و محتوای بیمارستان ها را مورد بررسی قرار دهد، وجود دارد.

Title: اثر درازمدت لوزارتان بر مهار تغییرات تعدادی و حجمی گلومرولهای کلیه در رتهای دیابتی – نفرکتومی یکطرفه (مطالعه استریولوژیک)

http://yafte.lums.ac.ir/article-1-946-fa.html

مقدمه: نفروپاتی دیابتی یکی از علل مرحله نهایی بیماری کلیوی است .به علت هیپرگلیسمی در کلیه آنژیوتانسین II بطور موضعی افزایش یافته و موجب القاء ضایعات کلیوی بویژه ضایعات گلومرولی می شود. دراین تحقیق گیرنده نوع1 آنژیوتانسینII(AT1)توسط لوزارتان بلوکه وتاثیر این دارو در مهار ضایعات کلیوی بطور کمی ارزیابی گردید. مواد و روشها: در این مطالعه تجربی 24 رت نر دوماهه نفرکتومی چپ گردیده و بطور تصادفی به سه گروه 8 تایی تقسیم شدند. سپس با تزریق آلوکسان(mg/Kg 120زیرجلدی)در گروههای دوم و سوم دیابت القاء گردید.5 روز بعد از القاء دیابت به گروه سوم مدت 8 هفته لوزارتان(mg/Kg/day 5)از راه دهان داده شد.سپس کلیه حیوانات تمام گروههابرداشته وفیکس گردید. هر کلیه به اسلایسهایی با ضخامت یک میلی متر بریده (نمونه گیری تصادفی سیستماتیک)وبعداز پردازش بافتی از هر اسلایس زوج برش با ضخامت 5 میکرون تهیه و با روش PAS رنگ آمیزی شدند. با استفاده از روش کاوالیری و دایسکتور فیزیکی حجم کلیه، حجم گلومرولهاو تعداد گلومرولها در هر کلیه برآورد گردید. تفاوت میانگینهای متغیرهادربین گروهها بانرم افزار SPSS12و روش آماری Mann withneyدر P<0.05 ارزیابی گردید. یافته ها: نتایج بدست آمده نشان دادند درمان با لوزارتان در گروه درمانی نسبت به گروه دیابتی بدون درمان در هر سه متغیر حجم کلیه،حجم گلومرولها وتعداد گلومرولها تفاوت معنی داری داشته است (05/0p<) وتوانسته است در مهار ضایعات مؤثر باشد. نتیجه گیری: مصرف لوزارتان در دز پایین و دراز مدت در رتهای دیابتی –نفرکتومی یکطرفه توانسته است بطور معنی داری در جلوگیری از افزایش حجم کلیه (حدود 84%) تأثیر داشته و افزایش حجم گلومرولی را 67% مهار ودر جلوگیری از کاهش تعداد گلومرولها حدود 68% مؤثر باشد.برای حصول نتایج بهتر ترکیب درمانی با داروهای دیگر پیشنهاد می گردد.

Title: لشمانیازیس احشایی درکودکان

http://yafte.lums.ac.ir/article-1-947-fa.html

مقدمه: لیشمانیوز احشایی بیماری مزمنی است که به صورت خاص کودکان زیر 5 سال را نوع L.Infantum وکودکان بزرگتر وبالغین جوان را درناحیه آفریقا وآسیا نوع (L.Donovani) مبتلا می کند. هدف از این مطالعه بررسی یافته های اپیدمیولوژی کلینیکوپاتولوژی , پاسخ درمانی بیماران مبتلا به لیشمانیوزیس ومقایسه آن با سایر مناطق آندمیک دنیا بود. مواد و روشها: این بررسی به صورت توصیفی صورت گرفت. 40 نفر از بیماران که با تشخیص لیشمانیوز احشایی درفاصله سالهای 1381-1379 دربیمارستان کودکان بستری شده بودند در این مطالعه وارد شدند. تشخیص بیماری به کمک بررسی مغز استخوان و تست الگوتیناسیون سیستم (DAT) مثبت صورت گرفته بود. افراد نمونه از نظر علائم بالینی تنها و یافته های آزمایشگاهی و روشهای درمانی مورد بررسی قرار گرفتند. یافته ها: در طی مدت 3 سال بررسی , از شهریور 79 لغایت شهریور 81 تعداد 40 مورد بیمار مبتلا به لیشمانیوز احشایی دراین مرکز بستری شده بودند، متوسط شیوع سنی 5/22 ماهگی بود. تب واسپلنومگالی درتمامی موارد , هپاتومگالی در29 مورد (5/72%) و ایکتر در8 مورد (20%) وجودداشت. 90% بیماران هموگلوبولین کمتر از10 داشتندو ترومبوسیتوپنی در 75% موارد مشاهده شد و بررسی مغز استخوان درتمام بیماران انجام شدکه در 5/92% موارد مثبت بود وتست DAT در 72% موارد مثبت گزارش شد. 3 بیمار با علائم هیپرتانسیون پورت به صورت ایکتروهپاتواسپلنومگالی مراجعه نموده بودند. تمامی بیماران تحت درمان با مگلومین آنتی مونات (گلوکانتیم) قرار گرفتند.فقط یک مورد فوت به علت عدم پاسخ به درمانهای مختلف و عود مکرر وجود داشت. نتیجه گیری: بیماری لیشمانیوز احشایی درسنین شیرخوارگی می تواند به صورت علائم درگیری کبدی خود را نشان دهد. این بیماری درمنطقه آذربایجان به درمان با آنتی موان پاسخ می دهد.داروهای گران قیمتی چون آمفوتریسین B باید برای موارد عود یا مقاوم به درمان در نظر گرفته شوند.

Title: تعیین باکتری‌های مسئول عفونت ادراری و حساسیت آنتی‌بیوتیکی آنها

http://yafte.lums.ac.ir/article-1-948-fa.html

مقدمه: عفونت ادراری یک بیماری شایع در سطح جامعه خصوصاً در نزد خانمها است. در غیاب کشت مثبت ادرار در اغلب موارد درمان عفونت ادراری تنها براساس وجود علائم بالینی و یافته های کامل ادرار صورت می گیرد. هدف از انجام این مطالعه تعیین سوشهای مسبب عفونتهای ادراری و الگوی حساسیت آنتی بیوتیکی هر کدام در شهر اهواز بود. مواد و روشها: این مطالعه گذشته نگر در طول سال 81 بر روی کلیه مراجعین به آزمایشگاه های خصوصی و دولتی اهواز انجام گرفت. از تمام مراجعین نمونه وسط ادرار استریل اخذ و در محیط های Blood Agar و Maconkey کشت داده شد و فراوانی کشت مثبت ادرار و حساسیت آنتی بیوتیکی پاتوژن های ادراری تعیین شد. یافته ها: از مجموع 48850 نمونه 34360 زن (34/70 %) و 14490 مرد (66/29%) مورد بررسی قرار گرفتند.از این تعداد 4106 (4/8%) کشت مثبت (3140 زن و 966 مرد) داشتند. شایعترین پاتوژن- های ادراری بترتیب شامل E.coli (24/46%) و استافیلوکوک ساپروفیتیکوس (8/25%) و کلبسیلا پنومونیه (93/16 %) بودند. انتروباکتر، پروتئوس، سیتروباکتر، پسودومونا و استافیلوکوک اپی درمیدیس، به ترتیب بقیه ارگانیسم های جدا شده را تشکیل می دادند.بیشترین حساسیت آنتی بیوتیکی در مورد سه جرم شایع بترتیب مربوط به نالیدیکسیک اسید، وانکومایسین و نیتروفورانتویین بود. نتیجه گیری: در مطالعه ما توزیع فراوانی پاتوژنهای ادراری و الگوی حساسیت آنتی بیوتیکی مطابق با کتب مرجع و گزارشات منتشر شده در دیگر شهرهای کشور بود.

Title: کارائی مدل دوجمله ای منفی با انباشتگی بیش از حد انتظار صفر در تحلیل مدت اقامت مادران پس از زایمان

http://yafte.lums.ac.ir/article-1-949-fa.html

مقدمه: زایمان مادران یکی از شایع ترین عوامل بستری شدن آنها در تمام کشورها است که مدل بندی آن می تواند چگونگی توزیع آن را تبیین نموده و عوامل مؤثر در افزایش یا کاهش آن را معین کند. هدف این مطالعه ارائه یک مدل مناسب جهت مدل بندی مدت زمان اقامت مادران در بیمارستان و مقایسه مدل های مختلف تبیین این مقادیر است. مواد و روشها: پژوهش حاضر یک مطالعه مشاهده ای ـ مقطعی بوده که بر روی یک نمونه تصادفی 1600 نفری از مادران مراجعه کننده به مراکز وابسته به دانشگاه علوم پزشکی اراک در نیمه اول سال 1383 جهت زایمان صورت گرفته است. از مادران انتخاب شده در نمونه مقادیر متغیر مدت اقامت آنها در بیمارستان(تعدادروزهایی که از پذیرش تا ترخیص در بیمارستان بوده اند) به عنوان متغیر پاسخ و متغیرهای سن مادر, توان دوم سن مادر, شغل مادر, فرزند ناهنجار متولد شده, رتبه تولد یا حاملگی, توان دوم رتبه تولد یا حاملگی, تعداد سقط های مادر, توان دوم تعداد سقط های مادر, تعداد فرزندان فعلی مادر, توان دوم تعداد فرزندان فعلی مادر, محل سکونت, نوع زایمان, وجود فرزند دوقلوی متولد شده و وجود فرزند سه قلوی متولد شده به عنوان متغیرهای مستقل در نظر گرفته شدند. جهت تحلیل داده ها از روشهای پیشرفته و جدید مدل بندی داده های شمارشی و روشهای ابتکاری مطرح شده در مقاله, استفاده شد. یافته ها: نتایج مدل بندی مقادیر مدت اقامت مادران نشان داد که در ابتدا مدل دو جمله ای منفی با توجه به اینکه مقادیر واریانس و میانگین متغیر پاسخ برابر نبودند, مدل مناسبی جهت تبیین مقادیر مدت اقامت مادران بوده است و متغیرهای وجود فرزند ناهنجار, نوع زایمان(طبیعی, سزارین) و دوقلو زائی متغیرهای معنادار آماری در این مدل بر روی مقادیرمدت زمان اقامت مادران بوده اند. در صورتیکه از مدل های اختصاصی تر جهت این مدل بندی استفاده شود (مدل های بریده شده در نقطه صفر پوآسنی و دوجمله ای منفی), مدل بکار برده شده بیشترمناسب است و متغیرهای سن, توان دوم سن, وجود فرزند ناهنجار, نوع زایمان, دوقلو زائی و سه قلو زائی متغیرهای معنادار در تبیین مقادیر مدت اقامت مادران بوده اند. نتیجه گیری: در مقاله حاضر با توجه به ارائه یک تغییر متغیر ساده بر روی مقادیر مدت اقامت مادران, یک مدل مناسب آماری جهت تبیین آنها و مدل بندی این مقادیر بدست آمد. مدل بدست آمده متغیر های بیشتری را با توجه به اختصاصی تر بودن این مدل نسبت به مدل های رایج ارائه نمود.

Title: بررسی سطح HBs Ab در اینترنهای واکسینه شده دانشگاه علوم پزشکی لرستان در پاییز 83

http://yafte.lums.ac.ir/article-1-950-fa.html

مقدمه: پرسنل بهداشتی و دانشجویان پزشکی در مقایسه با سایر گروههای جامعه به دلیل مواجهه مکرر با بیماران و فرآورده های خونی و نیز ترشحات قسمت های مختلف بدن در ریسک بالاتر ابتلاء به بیماریهای عفونی از جمله هپاتیت B قرار دارند (1). یکی از راههای پیشگیری، مصون سازی توسط واکسیناسیون علیه این بیماریها است. هدف از این مطالعه، تعیین سطح آنتی بادی ضد ویروس هپاتیت B پس از واکسیناسیون بود (1). مواد و روشها: این مطالعه توصیفی ـ مقطعی بر روی تعداد 36 نفر از اینترن های دانشگاه علوم پزشکی لرستان که 3 دوز واکسن را به صورت کامل دریافت نموده و از آخرین زمان تزریق آنها حداکثر 6 سال گذشته است، انجام شد. نمونه های خون گرفته شده از لحاظ سطح آنتی بادی ضد ویروس هپاتیت B به طریقه الیزا بررسی شدند. یافته ها: نتایج حاکی از آن بود که 4/44 درصد اینترنها پاسخ ایمنی متوسط و در حد قابل قبول نسبت به واکسیناسیون داشته (MIU 10 MIU > HBs Ab > 100) و 6/55 درصد اینترنها از سطح ایمنی خوبی نسبت به واکسیناسیون برخوردار بودند (MIU < 100). عدم پاسخ به واکسیناسیون در هیچ کدام از اینترن ها مشاهده نشد (MIU 10 <). بین سطح آنتی بادی ضد هپاتیت B (HBs Ab)، گروه سنی، جنسیت، فاصله آخرین تزریق واکسن و نیز رعایت فواصل بین تزریق 3 دوز واکسن رابطه معنی داری بدست نیامد. نتیجه گیری:کل نتایج بیانگر پاسخ ایمنی مناسب و قابل قبول در اینترنهای تحت مطالعه نسبت به دریافت 3 دوز واکسن بود.

Title: مقایسه میزان بروز یووئیت بعد از عمل کاتاراکت و لنز گذاری داخل چشمی با استفاده از مواد ویسکوالاستیک (ژل متیل سلولز و هیلون) و هوا

http://yafte.lums.ac.ir/article-1-951-fa.html

مقدمه: عمل جراحی کاتاراکت شایعترین نوع جراحی چشمی است که کیفیت مناسب عمل به بهبود بینایی کمک خواهد کرد. شایعترین عارضه بعد از عمل کاتاراکت، یووئیت است که زمان بروز آن معمولاً اوایل هفته دوم تا اواخر هفته سوم بعد از عمل می باشد. هدف از انجام این مطالعه مقایسه میزان بروز یووئیت بعد از عمل کاتاراکت با استفاده از مواد ویسکوالاستیک و هوا بود. مواد و روشها: این کارآزمائی بالینی بر روی 280 نفر از بیماران کاندید کاتاراکت چشم انجام شد. بیماران به طور تصادفی به دو گروه A و B تقسیم و تحت عمل جراحی قرار گرفتند. در گروه A برای جایگذاری لنز در داخل کپسول از ژل استفاده شد و در گروه B از هوای معمولی اتاق عمل استفاده شد. کلیه بیماران از روز اول بعد از عمل تا هفته چهارم بعد از عمل به طور مداوم (هفته ای یک بار) ویزیت شدند. هر بار به بیماران توصیه می شد در صورت تاری دید، درد و یا قرمزی شدید چشم فوراً مراجعه نمایند. اطلاعات بدست آمده توسط نرم افزار SPSS تجزیه و تحلیل شدند. یافته ها: از کل بیماران مورد مطالعه 140 نفر زن و 140 نفر مرد بودند. دامنه سنی بیماران 110-20 سال بود. از مجموع 280 نفر که تحت مطالعه قرار گرفتند 33 نفر (78/11%) دچار یووئیت بعد از عمل شدند که میزان بروز یووئیت در گروه A (گروهی که از ژل استفاده می کردند) 85/12% و در گروه B (گروهی که از هوا استفاده می کردند) 71/10% بود. آزمون Z فیشر تفاوت معنی داری بین میزان بروز یووئیت در دو گروه نشان نداد. نتایج این مطالعه همچنین نشان داد که بین سایر عوارض عمل از قبیل فشار بالا که از 20، قرار نگرفتن لنز بیرون از کپسول در دو گروه تفاوت معنی دار وجود نداشت. نتیجه گیری: با توجه به نتایج به دست آمده بین میزان بروز یووئیت در گروهی که ژل استفاده می کردند در مقایسه با گروهی که از هوا استفاده می کردند تفاوت معنی داری وجود نداشت. بنابراین با توجه به هزینه بالای ژل و در دسترس بودن هوا، توصیه می شود که از هوا در جریان عمل کاتاراکت استفاده شود.

Title: کاربرد پمپ سانتریفوژی در نارسایی قلب راست ناشی از بالا بودن فشار خون

http://yafte.lums.ac.ir/article-1-952-fa.html

مقدمه: روش درمان ایده آل در نارسایی قلب راست ناشی از بالا بودن فشار خون شریان ریوی که به علت آریتمی بطنی عارضه دار ایجاد شده باشد، هنوز مشخص نشده است. هر چند، چندین گزارش موردی در مورد بیماران آماده پیوند قلب که از قلب مصنوعی چپ یا قلب مصنوعی دو بطنی به عنوان پل پیوند استفاده شده، در ژورنالها چاپ شده است؛ اما هنوز گزارشی در مورد استفاده از پمپ سانتریفوژی برای درمان نارسایی قلب راست همراه با آریتمی بطنی چاپ نشده است. معرفی مورد: بیمار زنی 51 ساله از نواحی روستایی کردستان بود که به علت تنگی شدید دریچه میترال و وجود لخته در دهلیز چپ، جهت تعویض دریچه و تخلیه لخته به این مرکز ارجاع شده بود. بعد از تعویض دریچه میترال و تخلیه لخته دهلیز چپ به علت بالا بودن فشار شریان ریوی (110 mmHg) بیمار با وجود کاربرد داروهای اینوتروپ با حداکثر دوز، از دستگاه گردش خون برون پیکیری جدا نشد و به این علت با تعبیه بالون پمپ، بیمار به بخش مراقبت های ویژه انتقال یافت. در بخش مراقبت های ویژه به علت کاهش شدید فشار خون و تاکی کاردی و فیبریلاسیون بطنی مقاوم به درمان، بطور مکرر کاردیوورژن گردید و سرانجام تصمیم به تعبیه پمپ سانتریفوژی برای حمایت از بطن راست نارسا گرفته شد. بعد از 2 روز بیمار از قلب مصنوعی مذکور جدا شد و 7 روز بعد بالون پمپ خارج گردید و با وجود نارسایی تنفسی و تراکئوستومی بیمار کاملاً بهبود یافت و مرخص شد. نتیجه گیری: هر چند نارسایی قلب راست ناشی از فشار خون شدید شریان ریوی یک عارضه شوم بعد از عمل جراحی قلب باز است؛ اما در این بیمار استفاده توام از بالون پمپ و بیو پمپ برای نارسایی قلب راست باعث بهبودی بیمار شد.
